# Reduced Graphene Oxide-Gold Nanoparticle Nanoframework as a Highly Selective Separation Material for Aflatoxins

**DOI:** 10.1038/s41598-017-15210-1

**Published:** 2017-11-03

**Authors:** Wenbo Guo, Lidong Wu, Kai Fan, Dongxia Nie, Weijing He, Junhua Yang, Zhihui Zhao, Zheng Han

**Affiliations:** 1 Institute for Agri-food Standards and Testing Technology, Shanghai Key Laboratory of Protected Horticultural Technology, Shanghai Academy of Agricultural Sciences, Shanghai, 201403 China; 2 Key Laboratory of Control of Quality and Safety for Aquatic Products, Ministry of Agriculture, Chinese Academy of Fishery Sciences, Beijing, 100141 China

## Abstract

Graphene-based materials have been studied in many applications, owing to the excellent electrical, mechanical, and thermal properties of graphene. In the current study, an environmentally friendly approach to the preparation of a reduced graphene oxide-gold nanoparticle (rGO-AuNP) nanocomposite was developed by using L-cysteine and vitamin C as reductants under mild reaction conditions. The rGO-AuNP material showed a highly selective separation ability for 6 naturally occurring aflatoxins, which are easily adsorbed onto traditional graphene materials but are difficult to be desorbed. The specificity of the nanocomposite was evaluated in the separation of 6 aflatoxin congeners (aflatoxin B1, aflatoxin B2, aflatoxin G1, aflatoxin G2, aflatoxin M1 and aflatoxin M2) from 23 other biotoxins (including, ochratoxin A, citrinin, and deoxynivalenol). The results indicated that this material was specific for separating aflatoxin congeners. The synthesized material was further validated by determining the recovery (77.6–105.0%), sensitivity (limit of detection in the range of 0.05–0.21 μg kg^−1^), and precision (1.5–11.8%), and was then successfully applied to the separation of aflatoxins from real-world maize, wheat and rice samples.

## Introduction

Graphene is a single atomic plane of graphite, which is sufficiently isolated from its environment to be considered free-standing^1,2^. Owing to its excellent chemical and thermal stability, good electrical conductivity and high specific surface area, graphene has become one of the most frequently studied nanomaterials during the past decade^3^. Although many studies have focused on the physics of the graphene surface^4,5^, its chemical properties still remain underutilized and unexplored^6,7^. Graphene can selectively adsorb various atoms and molecules (such as NO_2_ and K)^8^. Normally, weakly attached adsorbates act as donors or acceptors and primarily lead to changes in the carrier concentration, such that graphene remains highly conductive with a strong adsorption capacity^9^.

Metals adsorbed on graphene can form different types of structures and change the electronic behaviour of graphene, thus giving rise to interesting physical and chemical properties^10^. Gierz *et al*. have developed a chemical doping method to introduce holes into graphene with gold, bismuth, or antimony^11^. The change in the carrier concentration caused by the adsorption of gold or bismuth on the graphene surface makes the material an excellent candidate for use as a gas sensor or adsorption material^12^. One type of graphene hybrid material, magnetic Fe_3_O_4_/graphene oxide nanocomposite, has been used as a simple and efficient extraction and pre-concentration material for trace polycyclic aromatic hydrocarbons (PAHs) in water samples^13^.

The preparation methods for metal-graphene composites, such as covalent bonding^14,15^ and chemical precipitation^16^, are difficult to control, and they also require rigorous conditions. Self-assembly is an easily controlled and effective method for fabricating composites that consist of metal oxides and carbon-based materials. Moreover, the open structure and relatively high surface area of graphene make uniform loading of metal nanoparticles easier, but the irreversible aggregation of graphene sheets in solution by van der Waals and π-π stacking interactions leads to a high diffusion resistance, low colloidal stability, poor reliability and repeatability, and non-specificity^17^. The addition of a second component (noble metal nanoparticles) that acts as a nanospacer and conductor can increase the graphene interlayer distance, thus minimizing agglomeration and making both faces accessible. Among the noble metal nanoparticles, Au nanoparticles (AuNPs) are one of the most studied, owing to their remarkable surface chemical properties, high chemical stability, excellent catalytic activity, biocompatibility, easy and controllable preparation. Because of these superior properties, AuNPs were chosen over other metals to modify graphene.

Separation materials are known to play vital roles in the development of accurate detection methods, the preparation of reference materials and the isolation and detoxification of naturally occurring biotoxins^18,19^. As an eligible alternative separation material, a reduced graphene oxide-AuNP (rGO-AuNP) nanoframework can overcome the barriers that limit the potential of currently available traditional materials, owing to its excellent properties, which include its high specific surface area (theoretically 2630 m^2^ g^−1^), size exclusion effect and high throughput (flux, loading capacity, etc.). To assess the physical and chemical properties of rGO-AuNPs, the separation of six aflatoxins from other compounds in real-world agricultural samples was examined as a model system. Aflatoxins, a family of toxic secondary metabolites produced by *Aspergillus* fungi, can contaminate a variety of important foodstuffs, especially peanuts, wheat and maize. The six most important members, aflatoxin B1 (AFB1), aflatoxin B2 (AFB2), aflatoxin G1 (AFG1), aflatoxin G2 (AFG2), aflatoxin M1 (AFM1) and aflatoxin M2 (AFM2), have been classified in group I as human carcinogens by the International Agency for Research on Cancer (IARC). Therefore, a rapid, selective system for the separation of aflatoxins was developed on the basis of the new rGO-AuNP material in the current work. This methodology should be useful in monitoring and control programs of agricultural practices and crop management, as well as in food safety administration.

## Experimental Section

### Materials and Solutions

AFB1, AFB2, AFG1, AFG2, AFM1, AFM2, ochratoxin A (OTA), ochratoxin α (OTα), deoxynivalenol (DON), 3-acetyldeoxynivalenol (3-ADON), 15-acetyldeoxynivalenol (15-ADON), neosolaniol (NEO), fusarenon X (Fus X), citrinin (CIT), fumonisins B1 (FB1), fumonisins B2 (FB2), zearalenone (ZEN), zearalanone(ZAN), α-zearalenol (α-ZOL), β-zearalenol (β-ZOL), α-zearalanol (α-ZAL), and β-zearalanol (β-ZAL) were purchased from Romer Labs, Inc. (Union, MO, USA). Sterigmatocystin (STM), gliotoxin (GLI), cyclopiazonic acid (CPA), patulin (PAT), alternariol (AOH), alternariolmethyether (AME) and altenuisin (ALS) were purchased from Sigma-Aldrich (St. Louis, MO, USA). Acetonitrile, hexane, acetone, formic acid, chloroauric acid, cysteine and ammonium acetate were also obtained from Sigma-Aldrich. Milli-Q water (Millipore, Billerica, MA, USA) was used for all analyses.

A total of 40 samples, including 20 peanut samples, 10 wheat samples, and 10 maize samples, were purchased from bazaars in Shanghai, China.

### Apparatus

Transmission electron microscopy (TEM) images were obtained on a JEM-2100 (Akishima, Tokyo, Japan) instrument with an accelerating voltage of 300 kV. Scanning electron microscopy (SEM) images and energy dispersive X-ray (EDX) analyses were collected on an FEI Quanta 200 field emission electron microscope (Hillsborough, Oregon, USA) operated at 20 kV. Ultraviolet–visible (UV-vis) absorption spectra were obtained using a JENA2010 spectrophotometer (JENA, Thuringia, Germany). Aqueous suspensions of graphene and rGO-AuNPs (0.2 mg mL^−1^) were used as the UV-vis samples, and deionized water was selected as the reference. Fourier transform infrared (FT-IR) spectra were recorded on an AVATAR 360 spectrophotometer (Waltham, MA, USA). The zeta potentials of rGO and AuNPs were measured with a Zetasizer (Malvern, UK). Atomic force microscopy (AFM) was performed on a MultiMode 8 AFM (Bruker) operating in tapping mode. The Au concentration was measured with an Agilent 7500 inductively coupled plasma mass spectrometer (ICP-MS) (Santa Clara, CA, USA).

HPLC analyses were performed on a Waters ACQUITY UHPLC system (Milford, MA, USA). Chromatographic separation of the targeted analytes was performed on an Agilent Poroshell 120 EC-C_18_ column (100 × 3 mm, 2.7 μm) (Agilent) with a mobile phase of methanol (A) and water containing 5 mmol L^−1^ ammonium acetate (B). A linear gradient elution program was designed as follows: initial, 10% (B), 1 min, 30% (B), 6 min, 90% (B), 12 min, 90% (B), 6.5 min, 10% (B), 8 min, 10% (B). The flow rate was 0.4 mL min^−1^, and the injection volume was 5 μL.

A Waters XEVO TQ-S mass spectrometer operated in both positive electrospray ionization mode (ESI^+^) and negative electrospray ionization mode (ESI^-^) was utilized for MS/MS analysis with the following parameters: source temperature, 150 °C; desolvation temperature, 500 °C; capillary interface voltage, 2.5 kV; cone voltage, 25 V; nebulizing gas, 7.0 bar; desolvation gas flow rate, 1000 L h^−1^. Quantification of the six aflatoxins and other biotoxins was performed in multiple reaction monitoring (MRM) mode, and the conditions were optimized for each toxin during infusion (Table S1). Data were acquired and processed in MassLynx v4.1 and Targetlynx software (Waters).

### Preparation of rGO-Cys and rGO-AuNPs

GO was obtained through a ball milling approach. Graphite powder (2.0 g) and steel balls (60 g, diameter: 1 cm) were placed in a hardened steel vial inside a glove box. This vial was purged with high purity argon (99.999%) for 20 min before being sealed. The ball milling process was carried out at 450 rpm for 20 hours to yield GO^20^. Subsequently, 25 mg of GO was dispersed in 50 mL of deionized water in a round-bottom flask, and 20 mg of N,N’-dicyclohexylcarbodiimide (NDC) was added at room temperature before ultrasonication of the mixture for 30 min. A weakly alkaline NH_3·_H_2_O solution (8 mol L^−1^) and 29 mg of L-cysteine (Cys) were added to the 50 mL suspension of GO and NDC (0.5 mg mL^−1^) to adjust the pH to approximately 10. The mixture was heated at 80 °C for 24 hours and then centrifuged at 13,000 rpm for 5 min. The residues were rinsed with Milli-Q water three times to remove unmodified GO. Finally, the cysteine-modified reduced GO (rGO-Cys) was obtained.

AuNPs were prepared in solution according to a seed-induced growth method by reduction of chloroauric acid. An aliquot (2.5 mL) of vitamin C (4 mg mL^−1^) was added dropwise into 50 mL of a chloroauric acid solution (0.1 mg mL^−1^), which was rapidly stirred for 2 min. Subsequently, 0.5 mL of a sodium citrate solution (10 mg mL^−1^) was added to quench the chemical reaction. After rinsing and centrifuging of the resulting solid (13,000 rpm, 5 min) three times, AuNPs were obtained. Suspensions of rGO-Cys (1.5 mL) and AuNPs (0.5 mL) were mixed and stirred for 30 min at 25 °C, and the resulting material was centrifuged and rinsed five times to obtain the final nanomaterial (rGO-AuNPs).

### Sample Preparation

Ground samples (5.00 ± 0.01 g) were suspended in 25 mL of an acetonitrile/water solution (84/16, v/v), and the mixtures were shaken for 30 min. After centrifugation for 15 min at a speed of 4500 rpm, an aliquot (5 mL) of each supernatant was transferred to a 10-mL centrifuge tube and dried with nitrogen gas at 50 °C. The residues were dissolved in 3 mL of a 5% acetonitrile aqueous solution, and 15 mg of rGO-AuNPs was added into each tube. The mixtures were ultrasonicated for 10 min, vortexed for 6 min and poured into empty solid-phase extraction (SPE) cartridges (3 mL) with one polypropylene frit placed at the bottom. After drying under vacuum, the SPE cartridges were washed with 5 mL of n-hexane. The targeted aflatoxins were eluted with 10 mL of acetonitrile/formic acid/water (89/1/10, v/v/v). The eluate was dried under a soft stream of nitrogen gas at 50 °C. The residues were dissolved in 1 mL of acetonitrile/water solution containing 5 mmol L^−1^ ammonium acetate (20:80, v/v), and the resulting solutions were passed through a 0.22 μm nylon membrane in preparation for LC-MS/MS analysis.

### Validation

Calibration curves were constructed by plotting the response versus the concentration of analyte in blank matrices. The limit of detection (LOD) and the limit of quantification (LOQ), which were designated as the concentrations of analyte providing signal-to-noise (S/N) ratios of 3/1 and 10/1, respectively, were used to evaluate the sensitivity. The signal suppression/enhancement (SSE), which was calculated by comparing the slopes of the standard addition and standard calibration plots, was used to assess the matrix effects. The recoveries were measured in non-contaminated samples in quintuplicate (n = 5) by using the method of standard addition. The low, intermediate and high spiking levels of aflatoxins were 1 μg kg^−1^, 10 μg kg^−1^ and 50 μg kg^−1^, respectively. Intra- and inter-day precision experiments were also performed on non-contaminated samples in quintuplicate (n = 5) with the same spiked levels of aflatoxins. The relative standard deviations (RSDs) in the single day analysis were used to evaluate the intra-day precision, whereas the RSDs for five consecutive days were used to evaluate the inter-day precision.

### Statistical Analysis

Statistical correlations were analysed by variance (ANOVA) and *p*-values, which are commonly used to evaluate the statistical significance of various data, using the software SPSS Statistics 17 (SPSS, Inc., Chicago, IL, USA).

### Safety Considerations

Aflatoxins and other biotoxins are highly toxic. MSDS information for these chemicals must be consulted, and precautions must be taken when handling them (i.e., wearing gloves, protective goggles and a mask).

## Results and Discussion

### rGO and rGO-AuNPs Physical Characterization

**Figure 1:**
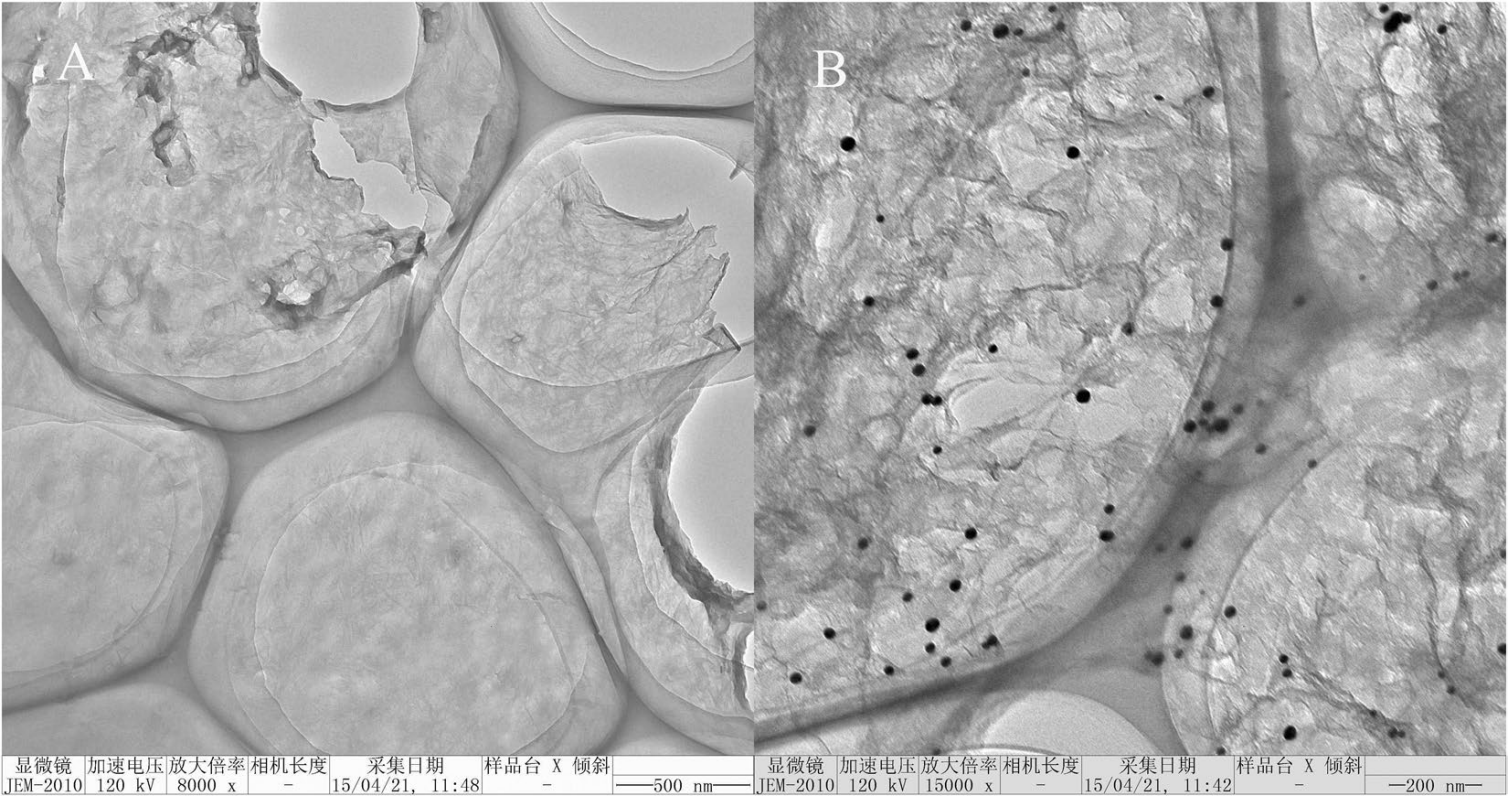
TEM images of GO (**A**) and rGO-AuNPs (**B**).

The structure and morphology of GO were characterized by TEM. Representative TEM images of GO and rGO-AuNPs are shown in Fig. 1. Figure 1A illustrates that high-quality, single-layer or few-layer GO was prepared, and its lateral dimension was approximately 1 µm. Figure 1B displays the TEM image of the rGO-AuNPs. Modification of the single-layer GO with cysteine provided numerous thiol-groups for the coordination of gold particles. The GO nanosheets were clearly formed, and AuNPs with an average particle size of approximately 20 nm were successfully anchored onto the surface of GO. The SEM images of GO and rGO-AuNPs are shown in Figure S1. In contrast to GO (Fig. S1A), the SEM image of rGO-AuNPs clearly showed that a stereo structure was formed (Fig. S1B). EDX analysis (Fig. S2) was also used to confirm the formation of the rGO-AuNP nanocomposite. The results showed the presence of cysteine, gold and carbon in rGO-AuNPs. The surface structure of rGO-AuNPs was visualized in the AFM image shown in Fig. S3. The AFM analysis further confirmed the uniform distribution of 20-nm diameter AuNPs anchored on the graphene net.

**Figure 2:**
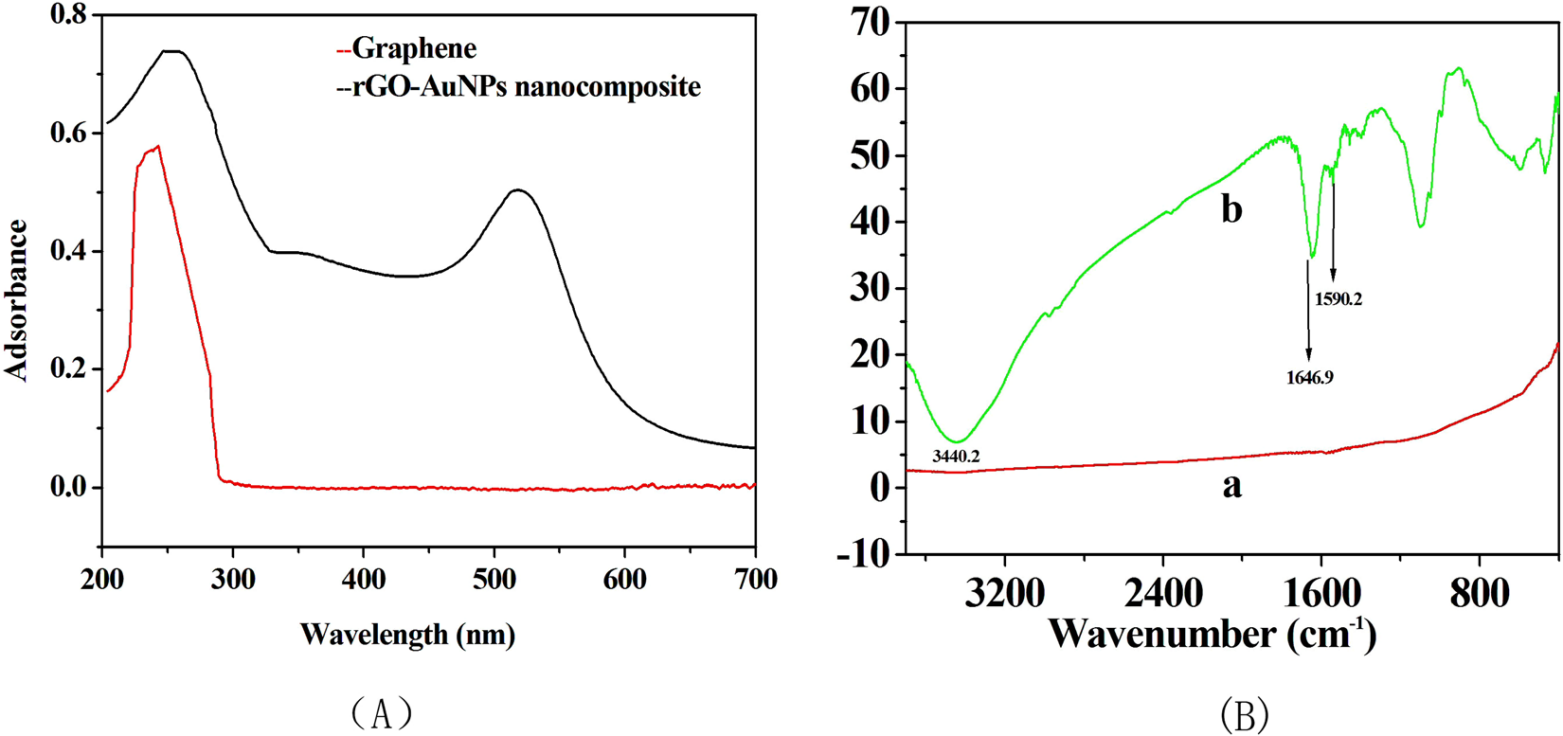
UV-vis absorbance spectra (**A**) and FT-IR spectra (**B**) of GO and rGO-AuNPs.

The rGO-AuNP nanocomposite was also characterized by UV-vis spectroscopy (Fig. 2A). The UV-vis absorption spectrum of GO is characterized by the π-π* transitions of the C=C plasmon peak at approximately 240 nm^21^. After reduction by cysteine and introduction of AuNPs, two peaks at approximately 260 and 520 nm were observed in the rGO-AuNP spectrum, and the plasmon peak redshifted to 270 nm, thus indicating that GO was reduced, and its aromatic structure was restored. Similar features have been reported by the Guo group^22^. The other peak at approximately 520 nm may be attributed to the typical surface-plasmon resonance band of the AuNPs (approximately 20 nm)^23^. The presence of this peak indicated that GO was successfully doped with AuNPs. The zeta potentials of GO and the AuNPs were −46.8 and 32.3 mV, respectively.

Figure 2B shows the FT-IR spectra of GO before and after introduction of the AuNPs. For GO, there were no obvious peaks (curve a). The curve of the L-cysteine-modified GO (curve not shown in Fig. 2B) was the same as that of rGO-AuNPs. Peaks for the oxygen functional groups of the nanomaterial were clearly observed. As shown in Fig. 2B (curve b), the broad and strong peak near 3440.2 cm^−1^ corresponded to the stretching vibrations of O–H in the carboxyl groups. In addition, the peaks at 1646 cm^−1^ and 1590 cm^−1^ arose from –NH bending vibrations and C=C stretching vibrations, respectively. Notably, the cysteine functional groups increased the surface hydrophilicity of the rGO-AuNP material.

## Optimization of Aflatoxin Separation by the Synthesized rGO-AuNPs

### Adsorption of Aflatoxins on rGO-AuNPs from Sample Matrices

**Figure 3:**
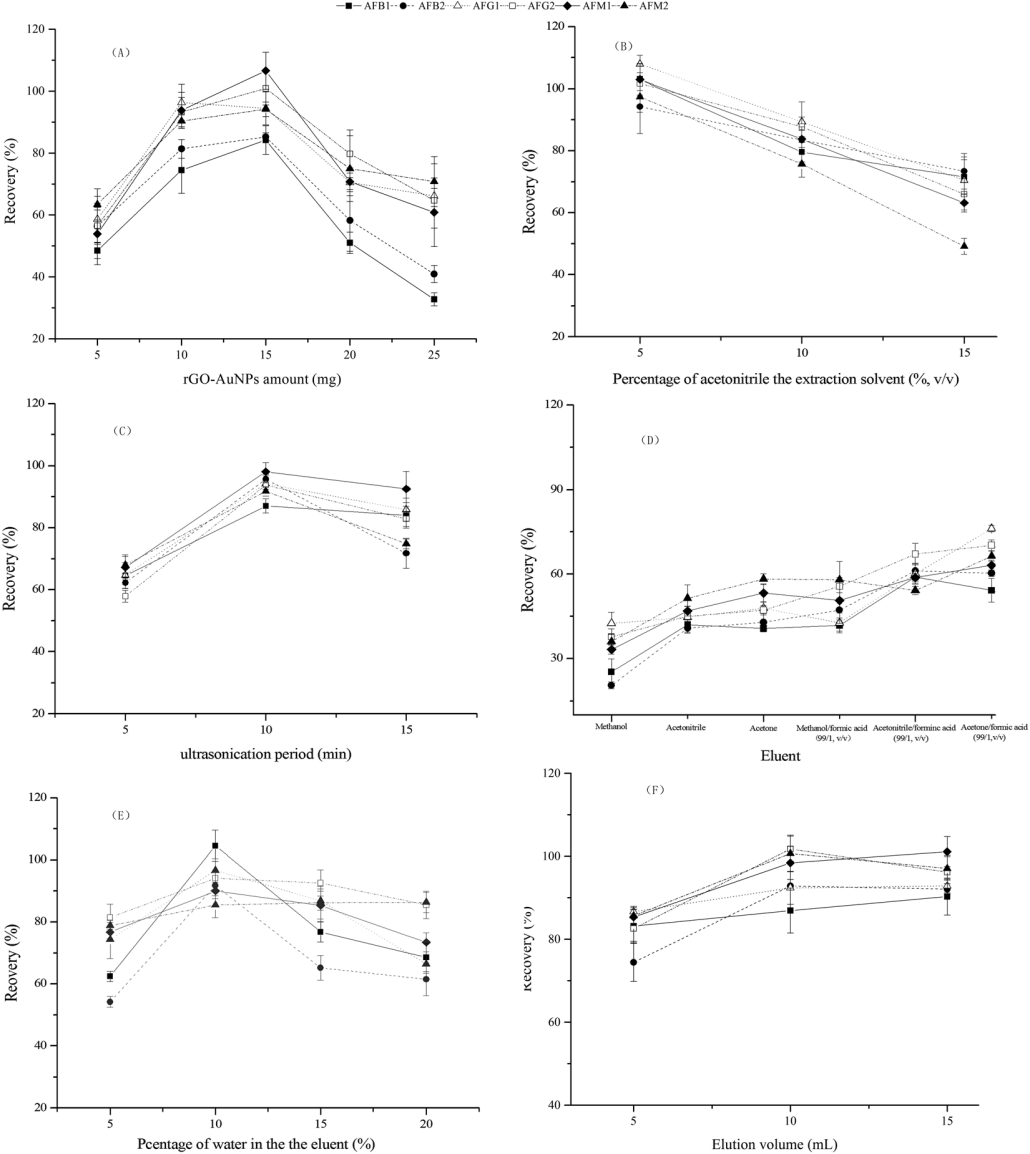
Effects of the key parameters on the aflatoxin separation performance of rGO-AuNPs, including the rGO-AuNPs amount (**A**), percentage of acetonitrile in the extraction solvent (**B**), ultrasonication period (**C**), eluent (**D**), percentage of water in the eluent (**E**) and elution volume (**F**).

To ensure that the targeted mycotoxins^24^ adsorbed to the prepared rGO-AuNPs from the sample matrices, several key parameters, including the amount of rGO-AuNPs, extraction solution, ultrasonication time and vortex time, were evaluated. As shown in Fig. 3A, the recoveries of the six mycotoxins (the chemical structures are shown in Fig. S4) dramatically increased from 48.5% to 106.6% when the amount of rGO-AuNPs was increased from 5 to 15 mg, but the recoveries sharply decreased when more than 15 mg of rGO-AuNPs was used. The differences in the recoveries were significant (*p* < 0.05) between group I (10 and 15 mg of rGO-AuNPs) and group II (5, 20 and 25 mg of rGO-AuNPs) for the six aflatoxins. For AFB1, a significant difference was also observed between 10 mg and 15 mg of rGO-AuNPs. There was clearly a balance point between the absorption and desorption of aflatoxins by rGO-AuNPs, and 15 mg was the balance point for the six aflatoxins. If the amount of rGO-AuNPs was below the balance point, the aflatoxins could not be fully absorbed on rGO-AuNPs. On the other hand, if the amount of rGO-AuNPs was too high, the aflatoxins could not be easily desorbed. The percentages of acetonitrile (5%, 10% and 15%) in the extraction solutions were then optimized for the separation of aflatoxins. As shown in Fig. 3B, the recoveries (94.2–107.9%) were acceptable for 5% acetonitrile. With increasing percentage of acetonitrile, the recoveries of all the targeted toxins clearly decreased, especially that of AFM2, for which nearly 30% was lost when 10% acetonitrile was used. Ultrasonication time ranging from 5 to 15 min was studied to optimize the dispersion of rGO-AuNPs in the sample solutions. When 5 min of ultrasonication was performed, the rGO-AuNP material formed aggregates, and consequently, unsatisfactory recoveries in the range of 57.9–68.0% were obtained (Fig. 3C). Satisfactory recoveries ranging from 87.1–98.1% were observed when the mixtures were ultrasonicated for 10 min. When the ultrasonication time was extended to 15 min, the recoveries decreased, especially those of AFB1 and AFB2, possibly because of strong adsorption of the targeted analytes on rGO-AuNPs, which could not be easily desorbed in the next steps. The differences in the recoveries were significant between 10 min and 5/15 min (*p* < 0.05) for all the aflatoxins except AFB1 and AFM1. In addition, vortex time from 3 to 9 min was also investigated and 6 min was selected because of the relatively higher recovery values (82.9–102.9%); however, there were no significant differences among the different vortex times (*p* > 0.1).

### Optimization of the Elution Solvent for the Selective Desorption of Aflatoxins from rGO-AuNPs

First, the elution efficiencies of three eluents, including methanol, acetonitrile and acetone, were tested, and unsatisfactory recoveries ranging from 20.6 to 67.1% were obtained. Then, 1% formic acid was added to improve the elution efficiency (Fig. 3D). Compared with the original solvents, the elution efficiency was slightly improved, and the highest recoveries were obtained in the range of 54.2–76.2% for acetone containing 1% formic acid, thus indicating that the adsorbent performance of rGO-AuNPs was affected by pH. The sample extractions and washing solutions were also analysed for aflatoxins, and no targeted analytes were detected in these solutions, thus indicating that the aflatoxins were adsorbed on the nanomaterial and could not be efficiently eluted. To further improve the elution efficiency, different percentages of water (5%, 10%, 15% and 20%) were added to the acid-acetone solution. As shown in Fig. 3E, the percentage of water in the solution significantly affected the elution efficiency. Satisfactory recoveries in the range of 85.5–104.5% were obtained when 10% water was added to the eluent, and the differences in the recoveries were significant (*p* < 0.05), especially for AFB1 and AFB2.

Different volumes of the eluent from 5 mL to 15 mL were also tested. As shown in Fig. 3F, 10 mL of acetone/water/formic acid (89:10:1, v/v/v) provided a satisfactory recovery ranging from 84.0 to 100.4%, whereas larger volumes of this eluent did not obviously increase the recovery, and there were no statistically significant differences among the different volumes of eluent (*p* > 0.1). On the basis of efficiency and economic considerations, 10 mL was selected as the optimum eluent volume.

### Evaluation of the Separation Efficiency of rGO-AuNPs

**Figure 4:**
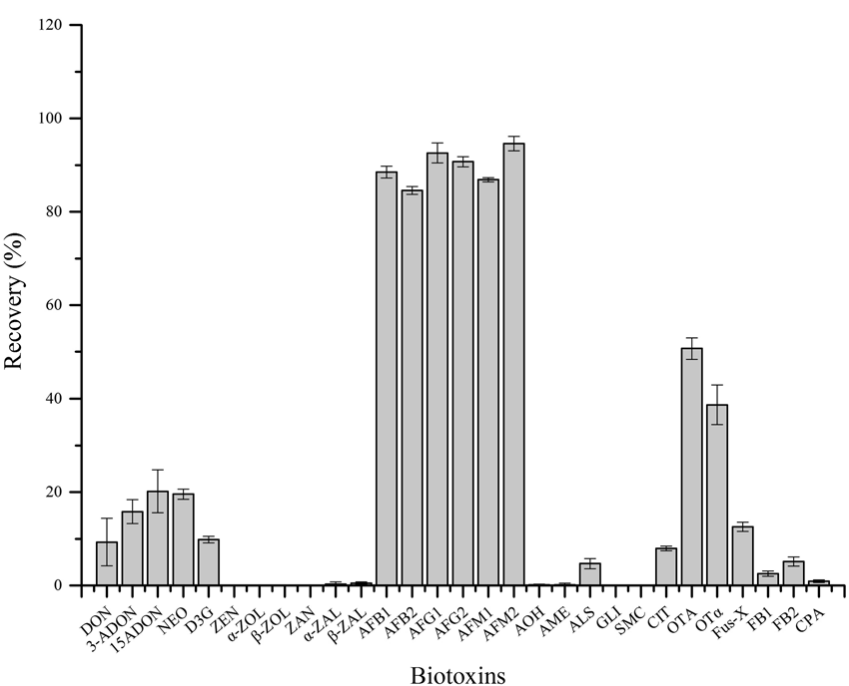
Selectivity of the synthesized rGO-AuNPs in the separation of various biotoxins.

The separation efficiencies of graphene and rGO-AuNPs were compared under the same parameters (amount, loading/extraction solutions, desorption solvent and volume) by using mixed standard solutions of the six aflatoxins (50 μg kg^−1^). As shown in Table S2, unsatisfactory recoveries in the range of 0.1–35.6% were obtained when graphene was used. Comparatively, higher recoveries in the range of 84.6–94.6% were obtained by using rGO-AuNPs. The selectivity of rGO-AuNPs was also tested in the separation of 29 naturally occurring biotoxins. As shown in Fig. 4, satisfactory recoveries were obtained for only the six aflatoxins, thus verifying the high selectivity of the synthesized material for aflatoxins. This selectivity may have been due to the special structure of rGO-AuNPs and its high surface hydrophilicity, which overcame the strong adsorption ability of graphene. In addition, AuNPs can modify the electron cloud density of the graphene aromatic ring structure and decrease the π-π* interactions between the aromatic ring structure of graphene and the aromatic rings of AFBs, AFMs and AFGs. The pore structure of rGO-AuNPs can provide numerous compound diffusion channels. All these excellent properties of rGO-AuNPs, as compared with those of graphene, lead to a better separation efficiency and selectivity.

Moreover, the performance of this method was compared with that of previously reported methods, in which aflatoxins were purified by MycoSep#226 Aflazon^+^ Columns^25^, ISOLUTE^(R)^ multimode SPE columns^26^, C-bonded silica^27^ and rGO-AuNPs, as summarized in Table S3. The synthesized rGO-AuNP material should be selected over the other sorbents because its performance was similar to or even better than those of the other methods, showing satisfactory recoveries and higher sensitivities, and requiring only 15 mg of the nanocomposite and substantially lower cost.

**Figure 5:**
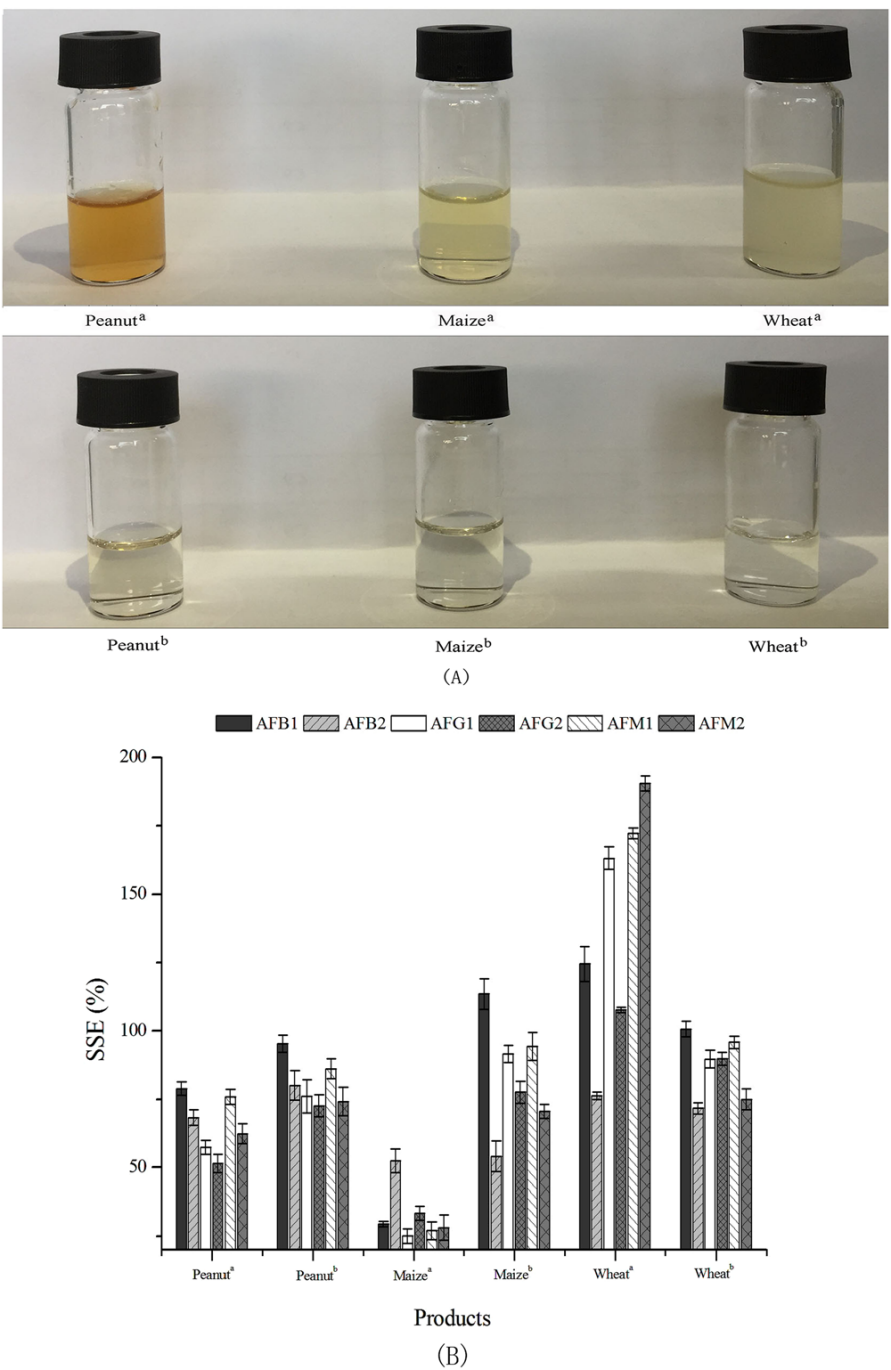
Visual appearance (**A**) and matrix effects (**B**) of six aflatoxins in peanut, maize and wheat matrices with and without treatment by rGO-AuNPs. ^a^Without treatment by rGO-AuNPs; ^b^Treated with rGO-AuNPs.

The aflatoxins separation efficiency of rGO-AuNPs in real-world peanut, wheat and maize samples was also investigated. The sample solutions treated with rGO-AuNPs were colourless and transparent (Fig. 5A), and the matrix effects were significantly lower than those observed in the experiments without using this synthesized material (Fig. 5B), thus indicating that rGO-AuNPs selectively separated aflatoxins from real-world sample matrices and eliminated interferences, e.g., pigment and tannin.

### Method validation

Good linearity was obtained for the six aflatoxins with correlation coefficients (R^2^) of more than 0.99 over the concentration range of 0.1–100 μg kg^−1^ in maize, wheat and peanut matrices. The values of LOD and LOQ were in the range of 0.05–0.21 μg kg^−1^ and 0.15–0.71 μg kg^−1^, respectively (Table S4). Satisfactory recoveries were obtained with mean values of 85.9–105.0% for peanut, 78.3–96.6% for maize and 77.6–104.1% for wheat. The RSDs of the six aflatoxins ranged from 1.5 to 11.8% for intra-day precision and from 2.1 to 10.3% for inter-day precision (Table 1).

**Table 1:** Recovery and precision results of six aflatoxins in peanut, maize and wheat samples (n = 5).

Aflatoxin	Spiked level (μg kg^−1^)	Peanut			Maize			Wheat		
		Recovery Mean ± SD	Intra-day precision (RSD, %)	Inter-day precision (RSD, %)	Recovery Mean ± SD	Intra-day precision (RSD, %)	Inter-day precision (RSD, %)	Recovery Mean ± SD	Intra-day precision (RSD, %)	Inter-day precision (RSD, %)
AFB1	1	105.0 ± 5.7	1.5	3.5	86.9 ± 1.4	5.2	3.1	87.8 ± 3.2	10.2	5.5
	10	86.0 ± 0.68	5.2	2.9	79.6 ± 8.0	5.1	5.0	78.4 ± 4.2	2.9	4.7
	50	102.17 ± 5.3	3.3	2.2	79.6 ± 1.8	2.9	4.0	104.1 ± 4.9	11.3	6.7
AFB2	1	101.5 ± 4.7	9.7	8.5	96.6 ± 1.5	8.9	7.6	81.7 ± 6.9	2.9	3.4
	10	93.1 ± 3.6	4.4	8.6	85.9 ± 5.9	10.4	10.1	88.4 ± 3.9	9.7	3.2
	50	96.7 ± 6.7	2.2	9.8	88.5 ± 6.5	5.8	6.1	96.7 ± 0.9	5.4	8.5
AFG1	1	104.0 ± 5.2	4.1	6.7	83.5 ± 3.0	2.8	6.4	95.9 ± 8.2	2.2	5.0
	10	94.7 ± 5.9	10.2	7.1	86.6 ± 3.5	5.1	3.7	80.9 ± 6.0	7.7	5.9
	50	99.8 ± 6.7	8.7	6.9	86.3 ± 6.0	2.8	3.2	89.2 ± 1.6	11.8	8.9
AFG2	1	86.2 ± 4.7	2.8	3.5	85.5 ± 0.5	5.5	4.6	98.6 ± 7.7	4.0	7.1
	10	92.6 ± 4.5	3.6	5.7	80.7 ± 4.4	8.7	6.9	84.6 ± 3.2	6.6	5.7
	50	100.2 ± 3.9	6.8	2.1	87.8 ± 3.1	9.6	5.2	90.5 ± 1.6	3.9	10.2
AFM1	1	101.0 ± 2.3	7.5	8.9	78.9 ± 2.8	3.5	3.3	103.9 ± 6.5	2.8	5.7
	10	89.6 ± 1.9	7.0	3.9	80.6 ± 2.5	4.8	5.4	79.9 ± 4.3	8.4	10.3
	50	93.0 ± 6.1	5.4	3.1	82.0 ± 2.3	2.9	5.7	91.6 ± 1.1	5.3	3.7
AFM2	1	97.5 ± 1.4	2.8	5.6	87.9 ± 5.5	11.4	9.6	88.1 ± 5.5	9.1	2.1
	10	87. ± 7.5	1.9	3.7	84.3 ± 2.6	8.6	4.5	77.6 ± 6.0	8.8	3.4
	50	85.9 ± 7.9	2.2	4.5	78.3 ± 4.1	5.6	7.2	77.7 ± 3.1	3.2	2.4

### Application

The contamination levels of aflatoxins in peanut, wheat and maize samples after separation by rGO-AuNPs and analysis by LC-MS/MS are shown in Table S4. AFB1 was the most frequently detected aflatoxin in the peanut, maize and wheat samples, with concentration ranges of 0.3–1.2 μg kg^−1^ (incidence of 70%), 1.7–83.3 μg kg^−1^ (incidence of 60%) and 0.6–1.0 μg kg^−1^ (incidence of 20%), respectively. These results are slightly lower than the previously reported values (incidence of 87.9% in peanuts) for samples from China^28^, possibly because of the investigation of samples from a different area with different environmental conditions. However, the determined contamination levels were nearly the same as those reported for samples from Brazil (incidence of 70% in peanuts)^29^. The other aflatoxins were rarely detected in the samples. Only AFB2 and AFM1 were detected in one peanut sample, with concentrations of 0.7 and 0.2 μg kg^−1^, respectively (Table S5). The contamination of AFM1 in real-world peanut samples indicated that AFM1 is not just a carcinogenic metabolite of AFB1 in milk and milk products, but it might also be a natural contaminant, as supported by previous findings^28,29^. The results clearly demonstrated that the examined crops are favourable matrices for the production of aflatoxins by *Aspergillus* species, and they emphasized the necessity of the synthesized material, which may be further used to improve the control of aflatoxins in China.

## Conclusions

The preparation and validation of an rGO-AuNP nanocomposite as a new type of carbon material were thoroughly investigated, and this material was found to be suitable for applications in the separation of aflatoxins. Owing to the special structure of the material and the introduction of AuNPs, the rGO-AuNP nanocomposite exhibited new properties, which address the strong adsorption and weak desorption of graphene. This material was successfully used for the selective separation of six aflatoxins from 29 biotoxins in real-world peanut, wheat and maize samples. This study provides a novel strategy for the preparation of an rGO-AuNP carbon material with new chemical and physical properties, which possesses broad application prospects in the fields of analytical and food chemistry.

## Electronic supplementary material

Supplementary information
