# Supplementary material for: Reduced Graphene Oxide-Gold Nanoparticle Nanoframework as a Highly Selective Separation Material for Aflatoxins
Source: Sci Rep. 2017 Nov 3;7:14484. doi: 10.1038/s41598-017-15210-1 (PMC5670127; doi:10.1038/s41598-017-15210-1)
Supplement: Supplementary file 1 — Supplementary information [file 41598_2017_15210_MOESM1_ESM.doc]

**Supplementary information:**

Reduced Graphene Oxide-Gold Nanoparticle Nanoframework as a Highly Selective Separation Material for Aflatoxins

Wenbo Guo ^1,a^, Lidong Wu ^2,a^, Kai Fan ^1^, Dongxia Nie ^1^, Weijing He ^1^, Junhua Yang ^1^, Zhihui Zhao ^1^, Zheng Han^1^,*

^1^ Institute for Agri-food Standards and Testing Technology, Shanghai Academy of Agricultural Sciences, Shanghai 201403, China

^2^ Key Laboratory of Control of Quality and Safety for Aquatic Products, Ministry of Agriculture, Chinese Academy of Fishery Sciences, Beijing 100141, China

^a^ Co-first author. Both authors contributed equally to this work.

* Corresponding author

Tel.: +86-21-62203612; Fax: +86-21-62203612;

E-mail address: [hanzheng_ok@163.com](mailto:hanzheng_ok@163.com)

**Table of Contents**

**Table S1.** MRM parameters of six aflatoxins and 23 other biotoxins.

**Table S2.** Comparison of separation efficiencies of graphene and rGO-AuNPs for aflatoxins.

**Table S3.** Comparative data of the present method with other solid-phase extraction approaches and HPLC analysis of aflatoxins in peanut sample.

**Table S4.** Linearity and sensitivity of six aflations in peanut, maize and wheat matrices.

**Table S5.** Contamination levels of six aflatoins in peanut, maize and wheat samples.

**Fig. S1** SEM images of rGO (A) and rGO-AuNPs (B).

**Fig. S2** EDX analysis of rGO-AuNPs.

**Fig. S3** AFM of rGO-AuNPs.

**Fig. S4** Chemical structures of six aflatoxins.

**Table S1. MRM parameters of six aflatoxins and 23 other biotoxins.**

| Mycotoxins | Retention time  (min) | ESI  (+/-) | Precursor ion  (m/z) | Cone voltage  (V) | Product ions  (m/z) | Collision energy  (eV) |
| --- | --- | --- | --- | --- | --- | --- |
| AFB1 | 5.27 | + | 313.2 | 74 | 285.0^a^ | 22 |
|  |  |  |  |  | 241.2^b^ | 36 |
| AFB2 | 5.11 | + | 315.2 | 2 | 287.1^a^ | 26 |
|  |  |  |  |  | 259.1^b^ | 28 |
| AFG1 | 4.91 | + | 329.2 | 12 | 243.1^a^ | 26 |
|  |  |  |  |  | 199.9^b^ | 42 |
| AFG2 | 4.73 | + | 331.2 | 22 | 245.1^a^ | 30 |
|  |  |  |  |  | 189.1^b^ | 40 |
| AFM1 | 4.76 | + | 329.2 | 40 | 273.1^a^ | 22 |
|  |  |  |  |  | 229.2^b^ | 38 |
| AFM2 | 4.53 | + | 331.2 | 4 | 273.1^a^ | 22 |
|  |  |  |  |  | 285.1^b^ | 24 |
| OTA | 5.84 | + | 403.8 | 4 | 239.0 ^a^ | 22 |
|  |  |  |  |  | 358.1 ^b^ | 12 |
| OTα | 4.72 | + | 257.2 | 2 | 193.1 ^a^ | 40 |
|  |  |  |  |  | 102.0 ^b^ | 28 |
| DON | 3.36 | - | 297.1 | 30 | 231.1 ^a^ | 13 |
|  |  |  |  |  | 221.0 ^b^ | 22 |
| 3-ACDON | 4.57 | + | 339.2 | 30 | 231.1 ^a^ | 12 |
|  |  |  |  |  | 137.0 ^b^ | 15 |
| 15-ACDON | 4.53 | + | 339.2 | 30 | 261.1 ^a^ | 12 |
|  |  |  |  |  | 279.1 ^b^ | 10 |
| NEO | 3.98 | + | 400.4 | 4 | 185.1 ^a^ | 20 |
|  |  |  |  |  | 305.2 ^b^ | 12 |
| Fus-X | 3.89 | - | 413.2 | 20 | 353.1 ^a^ | 10 |
|  |  |  |  |  | 263.1 ^b^ | 14 |
| CIT | 5.52 | + | 250.9 | 34 | 233.1 ^a^ | 16 |
|  |  |  |  |  | 191.1 ^b^ | 24 |
| FB1 | 5.90 | + | 722.5 | 64 | 352.4 ^a^ | 36 |
|  |  |  |  |  | 334.3 ^b^ | 40 |
| FB2 | 6.43 | + | 706.5 | 64 | 354.4 ^a^ | 34 |
|  |  |  |  |  | 318.4 ^b^ | 36 |
| ZEN | 6.57 | - | 319.2 | 66 | 175.1 ^a^ | 24 |
|  |  |  |  |  | 131.1 ^b^ | 30 |
| ZAN | 6.57 | - | 319.2 | 2 | 205.2 ^a^ | 24 |
|  |  |  |  |  | 163.5 ^b^ | 26 |
| α-ZOL | 6.51 | - | 319.2 | 2 | 159.9 ^a^ | 30 |
|  |  |  |  |  | 130.1 ^b^ | 34 |
| β-ZOL | 6.2 | - | 319.2 | 2 | 159.9 ^a^ | 30 |
|  |  |  |  |  | 130.1 ^b^ | 34 |
| α-ZAL | 6.40 | - | 321.2 | 2 | 259.2 ^a^ | 24 |
|  |  |  |  |  | 161.2 ^b^ | 28 |
| β-ZAL | 6.05 | - | 321.2 | 2 | 259.2 ^a^ | 24 |
|  |  |  |  |  | 161.2 ^b^ | 28 |
| STM | 6.85 | + | 325.2 | 72 | 310.1 ^a^ | 24 |
|  |  |  |  |  | 253.1 ^b^ | 44 |
| GLI | 5.46 | + | 326.8 | 4 | 263.1 ^a^ | 10 |
|  |  |  |  |  | 111.1 ^b^ | 22 |
| CPA | 5.67 | - | 335.2 | 82 | 140.1 ^a^ | 28 |
|  |  |  |  |  | 180.1 ^b^ | 26 |
| PAT | 2.67 | - | 153.0 | 2 | 81.1 ^a^ | 12 |
|  |  |  |  |  | 53.1 ^b^ | 18 |
| AOH | 5.91 | + | 259.0 | 64 | 213.1 ^a^ | 24 |
|  |  |  |  |  | 185.1 ^b^ | 28 |
| AME | 6.91 | + | 273.0 | 54 | 258.0 ^a^ | 25 |
|  |  |  |  |  | 128.1 ^b^ | 26 |
| ALS | 4.79 | - | 288.8 | 8 | 230.1 ^a^ | 16 |
|  |  |  |  |  | 240.1 ^b^ | 20 |

^a^: Quantitative ion; ^b^: Confirmation ion.

**Table S2. Comparison of separation efficiencies of graphene and rGO-AuNPs for aflatoxins.**

| Aflatoxin | Graphene | rGO-AuNPs |
| --- | --- | --- |
|  | Recovery  Mean±SD | Recovery  Mean±SD |
| AFB1 | 0.1±0.2 | 88.5±1.3 |
| AFB2  AFG1 | 4.5±1.3 | 84.6±0.9 |
|  | 3.2±1.5 | 92.6±2.2 |
| AFG2  AFM1 | 14.6±0.6 | 90.7±1.1 |
|  | 19.4±1.7 | 86.9±0.5 |
| AFM2 | 35.6±2.6 | 94.6±1.6 |

**Table S3. Comparative data of the present method with other solid-phase extraction approaches and HPLC analysis of aflatoxins in peanut sample.**

| Methods | Sample |  | | | Extraction sorbent | LODs  (μg kg^-1^) | | | | Refs | |  | | |
| --- | --- | --- | --- | --- | --- | --- | --- | --- | --- | --- | --- | --- | --- | --- |
| SPE-HPLC-UV | peanut | | | MycoSep#226 Aflazon+Column | | | 0.19-0.32 | | | | [^26^](#_ENREF_1) | |  |  |
| SPE-HPLC-FLD | peanut | | ISOLUTE(R) multimode SPE columns | | | | 0.25-0.68 | | | [^27^](#_ENREF_2) | |  | | |
| SPE-HPLC-FLD | Peanut | | C bonded silica | | | | 0.04-0.75 | | | [^28^](#_ENREF_3) | |  | | |
| HPLC-FLD coupled with GO | Peanut | | GO | | | | 0.08-0.65 | | | [^29^](#_ENREF_4) | |  | | |
| HPLC-MS/MS coupled with rGO-AuNPs | peanut | | rGO-AuNPs | | |  | | 0.05-0.21 | This work | | | | | |

**Table S4.** Linearity and sensitivity of six aflations in peanut, maize and wheat matrices.

| Matrix | Aflatoxin | Linear range  (μg kg^-1^) | Slope | Intercept |  | | R^2^ | Sensitivity | |
| --- | --- | --- | --- | --- | --- | --- | --- | --- | --- |
|  |  |  | (Mean±SD) | (Mean±SD) |  |  |  | LOD  (μg kg^-1^) | LOQ  (μg kg^-1^) |
| Peanut | AFB1 | 0.1-100 | 1299.0±20.9 | 628.6±10.4 | | 0.998 | | 0.07 | 0.25 |
|  | AFB2 | 0.1-100 | 5138.1±8.7 | 132.1±4.7 | | 0.996 | | 0.06 | 0.23 |
|  | AFG1 | 0.1-100 | 4542.5±54.7 | -234.8±24.2 | | 0.996 | | 0.05 | 0.17 |
|  | AFG2 | 0.1-100 | 1848.1±22.8 | 19.4±9.9 | | 0.993 | | 0.21 | 0.71 |
|  | AFM1 | 0.1-100 | 5869.1±32.8 | -169.3±7.8 | | 0.997 | | 0.05 | 0.15 |
|  | AFM2 | 0.1-100 | 3039.4±9.2 | -33.4±18.4 | | 0.998 | | 0.09 | 0.30 |
| Maize | AFB1 | 0.1-100 | 379.7±3.1 | 94.9±4.1 | | 0.991 | | 0.10 | 0.35 |
|  | AFB2 | 0.1-100 | 3209.1±160.7 | -202.0±18.4 | | 0.992 | | 0.06 | 0.21 |
|  | AFG1 | 0.1-100 | 1308.1±9.5 | -95.1±8.2 | | 0.994 | | 0.11 | 0.38 |
|  | AFG2 | 0.1-100 | 864.7±6.0 | -26.9±8.3 | | 0.995 | | 0.17 | 0.56 |
|  | AFM1 | 0.1-100 | 1830.1±26.5 | -126.9±13.2 | | 0.992 | | 0.07 | 0.22 |
|  | AFM2 | 0.1-100 | 1154.0±44.8 | 85.7±3.5 | | 0.997 | | 0.05 | 0.16 |
| Wheat | AFB1 | 0.1-100 | 464.1±9.5 | 96.7±6.1 | | 0.991 | | 0.16 | 0.55 |
|  | AFB2 | 0.1-100 | 4186.8±67.9 | -1268.3±28.9 | | 0.994 | | 0.11 | 0.38 |
|  | AFG1 | 0.1-100 | 1473.8±33.8 | -20.6±4.6 | | 0.995 | | 0.06 | 0.21 |
|  | AFG2 | 0.1-100 | 958.0±15.7 | 211.2±23.6 | | 0.991 | | 0.05 | 0.19 |
|  | AFM1 | 0.1-100 | 1902.6±7.7 | 76.0±8.4 | | 0.996 | | 0.07 | 0.24 |
|  | AFM2 | 0.1-100 | 1225.1±35.2 | -46.9±11.7 | | 0.998 | | 0.16 | 0.52 |

**Table S5. Contamination levels of six aflatoins in peanut, maize and wheat samples.**

| Aflatoxin | Peanut | |  | Maize | |  | Wheat | |  |
| --- | --- | --- | --- | --- | --- | --- | --- | --- | --- |
|  | Positive/Total  Samples | Range  (μg kg^-1^) |  | Positive/Total Samples | Range  (μg kg^-1^) |  | Positive/Total  Samples | Range  (μg kg^-1^) |  |
| AFB1 | 14/20 | 0.3~1.2 | | 6/10 | 1.7~83.3 | | 2/10 | 0.6~1.0 | |
| AFB2 | 1/20 | 0.7 | | 0/10 | <LOQ | | 0/10 | nd |  |
| AFG1 | 1/20 | <LOQ | | 0/10 | nd | | 0/10 | nd |  |
| AFG2 | 0/20 | nd | | 0/10 | nd | | 0/10 | nd |  |
| AFM1 | 1/20 | 0.2 | | 0/10 | nd |  | 0/10 | nd |  |
| AFM2 | 0/20 | nd | | 0/10 | nd | | 0/10 | nd |  |


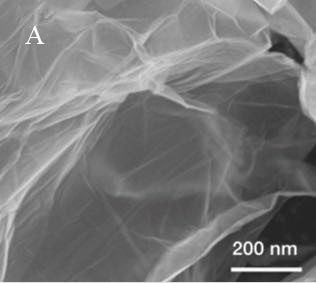

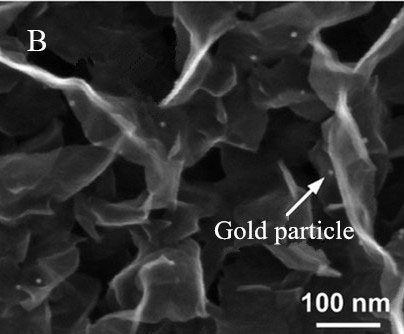


**Fig. S1** SEM images of rGO (A) and rGO-AuNPs (B).


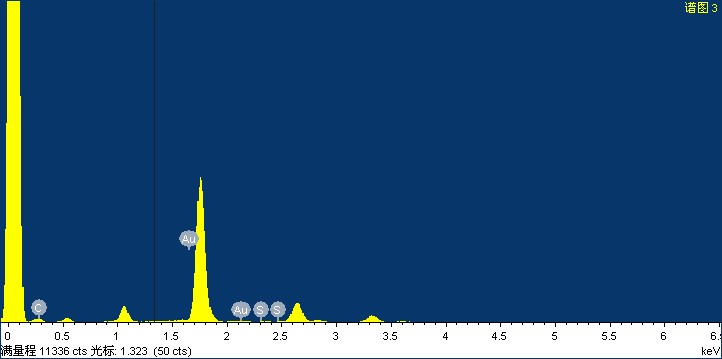


**Fig. S2** EDX analysis of rGO-AuNPs.


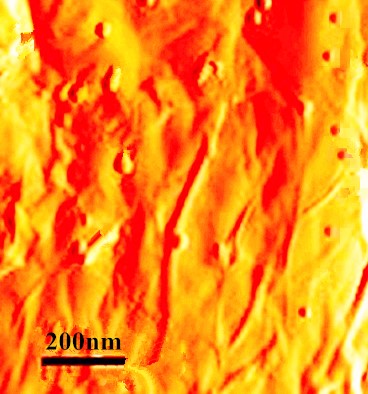


**Fig. S3** AFM of rGO-AuNPs.

**
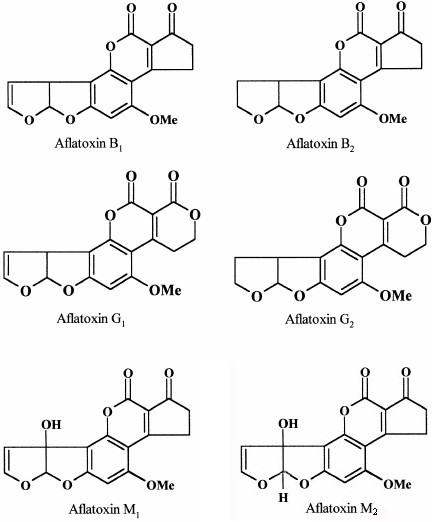
**

**Fig. S4** Chemical structures of six aflatoxins.
